# Upregulated MUC2 Is an Unfavorable Prognostic Indicator for Rectal Cancer Patients Undergoing Preoperative CCRT

**DOI:** 10.3390/jcm10143030

**Published:** 2021-07-07

**Authors:** Chia-Lin Chou, Tzu-Ju Chen, Yu-Feng Tian, Ti-Chun Chan, Cheng-Fa Yeh, Wan-Shan Li, Hsin-Hwa Tsai, Chien-Feng Li, Hong-Yue Lai

**Affiliations:** 1 Division of Colon and Rectal Surgery, Department of Surgery, Chi Mei Medical Center, Tainan 710, Taiwan; clchou3@gmail.com (C.-L.C.); van0112@hotmail.com (Y.-F.T.); 2 Department of Clinical Pathology, Chi Mei Medical Center, Tainan 710, Taiwan; a108n2@mail.chimei.org.tw (T.-J.C.); livelychord@yahoo.com.tw (H.-H.T.); 3 Department of Medical Technology, Chung Hwa University of Medical Technology, Tainan 717, Taiwan; wanshan0129@gmail.com; 4 Institute of Biomedical Sciences, National Sun Yat-Sen University, Kaohsiung 804, Taiwan; 5 Department of Medical Research, Chi Mei Medical Center, Tainan 710, Taiwan; h67350@gmail.com; 6 National Institute of Cancer Research, National Health Research Institutes, Tainan 704, Taiwan; 7 Department of Internal Medicine, Chi Mei Medical Center, Tainan 710, Taiwan; u802091@gmail.com; 8 Institute of Precision Medicine, National Sun Yat-Sen University, Kaohsiung 804, Taiwan; 9 Department of Pathology, School of Medicine, College of Medicine, Kaohsiung Medical University, Kaohsiung 807, Taiwan

**Keywords:** rectal cancer, chemoradiotherapy, MUC2, mucous barrier, treatment resistance

## Abstract

For locally advanced rectal cancer patients, introducing neoadjuvant concurrent chemoradiotherapy (CCRT) before radical resection allows tumor downstaging and increases the rate of anus retention. Since accurate staging before surgery and sensitivity prediction to CCRT remain challenging, a more precise genetic biomarker is urgently needed to enhance the management of such situations. The epithelial mucous barrier can protect the gut lumen, but aberrant mucin synthesis may defend against drug penetration. In this study, we focused on genes related to maintenance of gastrointestinal epithelium (GO: 0030277) and identified mucin 2 (*MUC2*) as the most significantly upregulated gene correlated with CCRT resistance through a public rectal cancer transcriptome dataset (GSE35452). We retrieved 172 records of rectal cancer patients undergoing CCRT accompanied by radical resection from our biobank. We also assessed the expression level of MUC2 using immunohistochemistry. The results showed that upregulated MUC2 immunoexpression was considerably correlated with the pre-CCRT and post-CCRT positive nodal status (*p* = 0.001 and *p* < 0.001), advanced pre-CCRT and post-CCRT tumor status (*p* = 0.022 and *p* < 0.001), vascular invasion (*p* = 0.015), and no or little response to CCRT (*p* = 0.006). Upregulated MUC2 immunoexpression was adversely prognostic for all three endpoints, disease-specific survival (DSS), local recurrence-free survival (LRFS), and metastasis-free survival (MeFS) (all *p* < 0.0001), at the univariate level. Moreover, upregulated MUC2 immunoexpression was an independent prognostic factor for worse DSS (*p* < 0.001), LRFS (*p* = 0.008), and MeFS (*p* = 0.003) at the multivariate level. Collectively, these results imply that upregulated MUC2 expression is characterized by a more advanced clinical course and treatment resistance in rectal cancer patients undergoing CCRT, revealing the potential prognostic utility of MUC2 expression.

## 1. Introduction

Colorectal cancer (CRC), starting from the large intestine (colon) or rectum, ranks third in terms of incidence and second in terms of mortality worldwide [1]. It is noteworthy that the rising incidence was driven by rectal cancer in Asia, especially in young patients (age < 50 years) who tend to present at a more advanced stage [2]. Benefitting from the development of total mesorectal excision, the prognosis has been improved for early-stage rectal cancer patients without lymph node metastasis. On the other hand, the introduction of neoadjuvant concurrent chemoradiotherapy (CCRT) before surgery allows tumor downstaging and increases the rate of sphincter conservation [3] for clinically staged T3/T4 or node-positive rectal cancer patients. However, the efficacy of preoperative CCRT varies among different individuals (and only approximately 20% of patients) achieve a pathologic complete response [4]. As patients who achieve a complete response do not necessarily need to undergo radical resection and have satisfactory outcomes, the identification of predictive biomarkers and therapeutic targets is critical for selecting better treatment strategies.

The dynamic crosstalk of tumors with their microenvironment consisting of immune cells, stromal cells, and the extracellular matrix (ECM) affects treatment efficacy and determines whether the primary tumor is eradicated or metastasizes. ECM remodeling shaped by reciprocal interactions between cells and the ECM is leveraged by tumors to promote tumorigenesis and metastasis. In the primary tumor, ECM stiffness characterized by lysyl oxidase (LOX)-mediated collagen crosslinking forms a physical barrier and influences drug penetration to tumor cells [5]. In addition, specific factors secreted from the tumor microenvironment recruit and activate bone marrow-derived cells to create a metastatic niche, collectively known as ECM degradation [6]. Consequently, a better comprehension of the molecular characterization of ECM stiffness and ECM degradation can provide clues for how such pathways be therapeutically targeted.

Mucin 2 (MUC2), a secreted gel-forming glycoprotein, is produced mainly by the intestines and is also found in the airways and urinary bladder. The human *MUC2* gene, mapped to chromosome 11p15.5, encodes an *O*-glycosylated mucin. The MUC2 proteins are catenated by disulfide bonds to form a high molecular weight mucous barrier and protect the gut lumen [7]. MUC2 expression is downregulated in patients with ulcerative colitis and Crohn’s disease [8]. Additionally, it has been reported that low MUC2 expression is prognostic of poor outcomes in CRC patients, but those who received radiation or chemotherapy were excluded [9,10]. Interestingly, high MUC2 expression is prognostic of worse survival in metastatic colon cancer patients treated with hyperthermic intraperitoneal chemotherapy following surgery [11]. Nevertheless, the correlations of MUC2 expression with the clinical outcomes of nonmetastatic rectal cancer patients undergoing preoperative CCRT are not well understood.

## 2. Patients and methods

### 2.1. Data Mining of a Public Transcriptome Dataset

To estimate the efficacy of preoperative CCRT, a public rectal cancer dataset (GSE35452) including 46 patients receiving CCRT followed by curative resection was utilized for transcriptomic profiling. In this dataset, biopsy specimens were collected during colonoscopic examination before CCRT. We computerized the raw CEL files with the statistical software Nexus Expression 3 (BioDiscovery, El Segundo, CA, USA) to quantify expression levels, and all probes were analyzed without preselection. Referring to the efficacy of CCRT, the specimens were separated into “responders” and “non-responders”, and a comparative analysis was conducted. We highlighted differentially expressed genes related to maintenance of gastrointestinal epithelium (GO: 0030277) and further chose those with a *p*-value less than 0.001 and expression fold change > ±1.5 log_2_ ratio for further analysis.

### 2.2. Patient Eligibility and Enrollment

Approved by the Institutional Review Board of Chi Mei Medical Center (10302014), this study was conducted on a total of 172 records of rectal cancer patients with formalin-fixed paraffin-embedded (FFPE) tissue specimens from the biobank. The primary clinical stage was determined via imaging tests, and only patients with T3/T4 disease or node positivity and without distant metastasis were eligible. All patients received a total dose of 45–50 Gy radiation concomitant with 5-fluorouracil (5-FU)-based chemotherapy before proctectomy. For patients presenting with a nodal status greater than N1 or a pre-CCRT or post-CCRT tumor status greater than T3, adjuvant chemotherapy was given. All patients were routinely monitored after diagnosis until death or the last follow-up.

### 2.3. Histopathological and Immunohistochemical Evaluations

To obtain more objective results, two independent pathologists (W.-S.L. and H.-L.H.) who were blinded to the clinical information of the patients reviewed all tumor specimens. The T and N stages were determined in accordance with the 7th American Joint Committee on Cancer (AJCC) TNM staging system. In concordance with the description by Dworak et al. [12], the tumor regression grade, which is predictive of the tumor response to CCRT, was evaluated. Immunohistochemical staining was performed in accordance with our previous study [13] and probed with an anti-MUC2 antibody. The H-score was applied to evaluate MUC2 immunoreactivity and was quantified with the following equation:

H-score = Σ*Pi* (*i* + 1) (1)

where *Pi* is the percentage of stained tumor cells for each intensity, varying from 0% to 100%, and *i* is the intensity of staining (0 to 3+). Formulated on a combination of the intensity and percentage of positively stained tumor cells, the H-score was generated and ranged from 100 to 400. H-scores above or identical to the median of all scored cases were determined as having high MUC2 expression.

### 2.4. Statistical Analysis

The chi-square (χ^2^) test was used to measure the correlations between clinicopathological features and MUC2 expression. Survival curves were plotted utilizing the Kaplan-Meier method, and the log-rank test was used to calculate and compare the time from the operation to death (or last seen alive) or the appearance of recurrence (or last seen relapse-free). Those factors with clinical significance in the univariate analysis were included in the Cox proportional hazard model for multivariate analysis. All statistical analyses were conducted using SPSS software version 22.0 (IBM Corporation, Armonk, NY, USA), and two-tailed tests with *p* < 0.05 were considered statistically significant.

## 3. Results

### 3.1. MUC2 Is Recognized as the Most Significant Differentially Expressed Gene Connected with Maintenance of Gastrointestinal Epithelium

**Figure 1:**
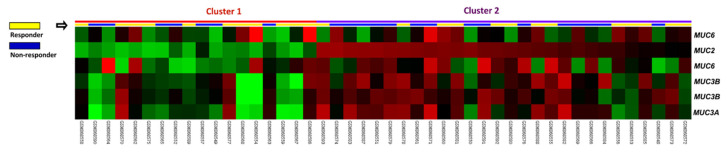
Expression profiling of genes associated with maintenance of gastrointestinal epithelium (GO: 0030277) in relation to the response to CCRT. We identified *MUC2* as the most significantly upregulated gene connected with maintenance of gastrointestinal epithelium among CCRT non-responders.

Recent studies have supported the development of genetic biomarkers for improving the stratification of risk and clinical outcomes. To predict the response to preoperative CCRT in rectal cancer patients, we examined prospective biomarkers by analyzing a public transcriptome dataset (GSE35452). Based on the response to CCRT, 24 patients (52.2%) were categorized as responders, while 22 patients (47.8%) were classified as non-responders. Focusing on maintenance of gastrointestinal epithelium (GO: 0030277), we identified six probes covering four transcripts: *MUC2*, *MUC3A*, *MUC3B*, and *MUC6* (Table 1 and Figure 1). We selected *MUC2* for further analysis, as its expression was considerably higher among CCRT non-responders (*p* = 0.0002). This discovery inspired us to further investigate the expression level and clinical relevance of *MUC2* in rectal adenocarcinoma.

### 3.2. Clinicopathological Features of Patients with Rectal Carcinoma in Our Cohort

A total of 172 records of rectal cancer patients were recovered from the biobank, and most patients were male (*n* = 108, 62.8%) and less than 70 years old (*n* = 106, 61.6%) (Table 2). The invasive depth of 81 patients (47.1%) was restricted to the muscularis propria (cT1-2), and there was no regional lymph node metastasis (cN0) in 125 patients (72.7%) during pre-CCRT clinical staging. After CCRT, the depth of invasion of 86 patients (50%) was pathologically beyond the muscularis propria (ypT3-4), and lymph node metastasis (ypN1-2) was found in 49 patients (28.5%). Vascular invasion was observed in 15 (8.7%) patients, and perineurial invasion was detected in 5 (2.9%) patients. The tumor regression grade was applied to predict the tumor response to CCRT, and the results showed that 37 patients (21.5%) had no or little response (grade 0–1), 118 patients (68.6%) had a moderate response (grade 2–3), and 17 patients (9.9%) had a complete response (grade 4).

### 3.3. Correlations of MUC2 Immunoexpression with Clinicopathological Parameters

**Figure 2:**
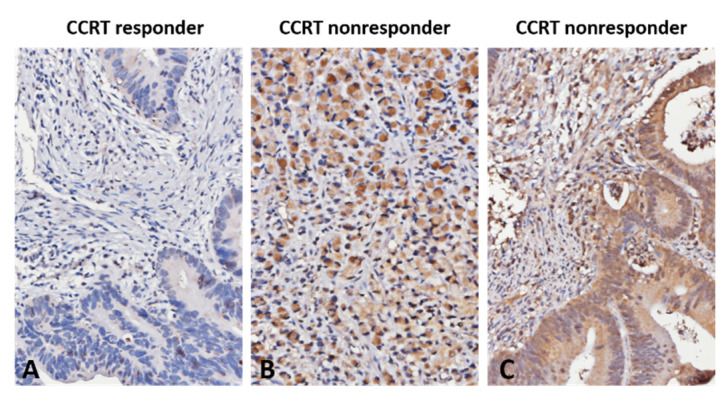
Immunohistochemical detection of MUC2. Representative images of rectal adenocarcinoma exhibiting high MUC2 immunoexpression among CCRT non-responders. (**A**) CCRT responder; (**B**) CCRT non-responder (signet-ring cell carcinoma); (**C**) CCRT non-responder (adenocarcinoma with ordinary glandular morphology).

Representative images of MUC2 immunohistochemical staining are displayed in Figure 2A–C. MUC2 immunoreactivity in CCRT nonresponsive rectal carcinoma was considerably higher than that in CCRT responsive rectal carcinoma. Table 2 exhibits the associations between MUC2 immunoexpression and its clinical relevance in rectal adenocarcinoma. Upregulated MUC2 expression was considerably connected with the pre-CCRT and post-CCRT positive nodal status (*p* = 0.001 and *p* < 0.001), advanced pre-CCRT and post-CCRT tumor status (*p* = 0.022 and *p* < 0.001), and vascular invasion (*p* = 0.015). Furthermore, tumors with high MUC2 expression were considerably connected with no or little response to CCRT (*p* = 0.006).

### 3.4. Survival Analyses and Clinical Implications of MUC2 Expression

**Figure 3:**
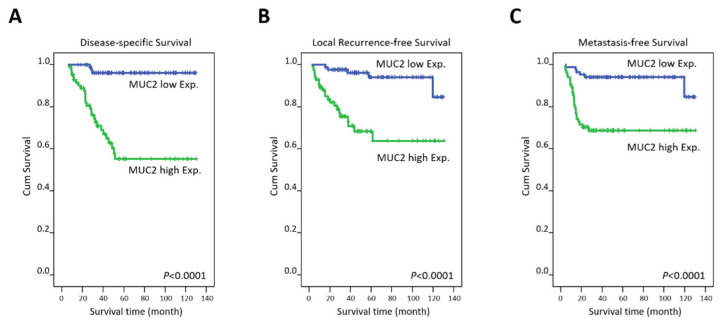
Survival analysis. Kaplan-Meier plots were generated and showed that high MUC2 immunoexpression was considerably connected with inferior (**A**) disease-specific survival; (**B**) local recurrence-free survival; and (**C**) metastasis-free survival.

Tumor specimens with high MUC2 expression were adversely prognostic for all three endpoints, disease-specific survival (DSS), local recurrence-free survival (LRFS), and metastasis-free survival (MeFS) (all *p* < 0.0001), at the univariate level (Table 3 and Figure 3A–C). A low degree of tumor regression and a progressive post-CCRT tumor status were also considerably connected with worse outcomes in all three endpoints (all *p* < 0.009). Pre-CCRT lymph node metastasis was considerably connected only with inferior LRFS (*p* = 0.007). Vascular invasion was prognostic for poor DSS and LRFS (*p* = 0.0184 and *p* = 0.0028). Furthermore, high MUC2 expression was independently prognostic for inferior DSS (*p* < 0.001), LRFS (*p* = 0.008), and MeFS (*p* = 0.003) in the multivariate analysis (Table 4). A low degree of tumor regression remained prognostically significant for worse LRFS (*p* = 0.037) and MeFS (*p* = 0.018).

## 4. Discussion

ECM stiffness, mainly determined by the amount of collagen and hyaluronan, can influence the tumor response to anticancer agents by forming a physical barrier. With structural similarity to von Willebrand factor (vWF), MUC2 can also bind to collagen or other connective tissue components, making cancer cells lose permeability to resist chemotherapy [7]. In addition, by using the Similarity Matrix of Proteins (SIMAP) database [14], we identified *MUC5AC* as one of the top similar genes (paralogs) for the *MUC2* gene. It has been suggested that the MUC5AC-CD44 axis promotes tumorigenesis and confers resistance to chemotherapy, including 5-FU, in CRC [15]. Mucinous adenocarcinoma, accounting for 10–15% of CRCs, is characterized by distinct molecular and clinicopathological features, including MUC2 overexpression, microsatellite instability (MSI), and multiple metastases [16]. Despite the fact that CRC patients with MSI have a good response to immune checkpoint inhibitors, it has been reported that mucinous CRC with MSI is correlated with low programmed death-ligand 1 (PD-L1) expression and a poor response to PD-L1 inhibitors [17]. On the other hand, the ECM protein MUC2 with heavily crowded *O*-glycans can mask immunodominant conformation and prevent neoepitope generation [18] and create a barrier to cytotoxic T cell infiltration [19]. Interestingly, MUC2 overexpression is not exclusive to mucinous CRC, as aberrantly overexpressed MUC2 has also been found in nonmucinous CRC [20]. Since most patients in our cohort had nonmucinous rectal cancer, whether high MUC2 expression is correlated with multiple metastases and an inferior response to immunotherapy in nonmucinous rectal cancer needs further investigation.

Tumor-associated macrophages, a class of immune cells, represent the major component of the tumor microenvironment and constitute a heterogeneous and plastic cell population, varying from a proinflammatory (M1-like) to an anti-inflammatory (M2-like) state. In response to interferon γ (IFNγ), lipopolysaccharide (LPS), tumor necrosis factor α (TNFα), and interleukin-12 (IL-12), macrophages can acquire the M1 phenotype, which governs the innate host defense and kills tumor cells in the context of Th1 immunity. In contrast, upon exposure to IL-4, IL-10, and IL-13, transforming growth factor beta 1 (TGFβ1), and prostaglandin E2 (PGE2), macrophages can undergo M2 activation, which is characterized by tissue repair, matrix remodeling, and tumor promotion, and mirrors those of Th2 responses. Shaped by bidirectional communication between cells and the ECM, obesity (a CRC risk factor)-associated ECM remodeling can also activate M2 macrophages [21]. In addition, cumulative evidence has demonstrated that M2 macrophages confer 5-FU resistance in CRC [22] and that in rectal cancer, CCRT non-responders are correlated with the M2 macrophage phenotype in the tumor microenvironment [23]. Interestingly, MUC2 overexpression is connected with M2 macrophage polarization and poor survival in ovarian cancer patients [24]. Furthermore, dendritic cells exposed to MUC2 produce less TNFα and IL-12, as well as more IL-10 and TGFβ1 in the large intestine [25], which suggests that MUC2 can deliver immunoregulatory signals to support tumor growth and treatment resistance. Although loss of the anti-inflammatory effects of MUC2 causes inflammatory bowel disease and CRC in the early stage, high MUC2 expression may affect treatment efficacy through specific factors secreted from the tumor microenvironment, which could partially underlie the complicated role of inflammation in cancer progression.

To further elucidate the roles of MUC2 in CRC, a gene coexpression network was analyzed, and the top 200 genes that were positively associated (Supplementary Table S1) or negatively associated (Supplementary Table S2) with *MUC2* from the Cancer Genome Atlas (TCGA) database (*n* = 594) were evaluated. Impressively, the regenerating family member 4 (*REG4*) gene was identified as the fourth most significantly positively correlated gene with *MUC2* (Spearman’s correlation: 0.776) (Supplementary Figure S1A) and one of the predictive factors associated with CCRT resistance in rectal cancer in our previous study [26]. Carbohydrate moieties can be utilized by cancer cells to escape recognition by immune cells. As a calcium-dependent lectin, REG4 can selectively recognize and bind glycan epitopes of glycoproteins or free carbohydrates. However, whether heavily glycosylated MUC2 can orchestrate REG4 to evade immune elimination needs further validation. In contrast, the epiregulin (*EREG*) gene was one of the top 200 genes that were co-downregulated with *MUC2* (Spearman’s correlation: −0.379) (Supplementary Figure S1B) and one of the favorable factors among rectal cancer patients receiving CCRT in our previous research [27]. Collectively, these results indicate that the molecular characterization of rectal cancer is complicated and interactive, and integration of these favorable and unfavorable biomarkers can more precisely guide treatment.

In addition, the PANTHER classification system revealed that the top two and three biological process terms that correlated with *MUC2* upregulation were maintenance of gastrointestinal epithelium (GO: 0030277, fold enrichment: 25.12) and epithelial structure maintenance (GO: 0010669, fold enrichment: 18.6), respectively (Supplementary Figure S2), further implying that MUC2 is functionally related to maintenance of the gastrointestinal epithelial structure. Numerous cytokines and neurotransmitters, including acetylcholine, have been shown to upregulate MUC2 expression [28]. Interestingly, in terms of molecular function, we identified neurotransmitter binding (GO: 0042165, fold enrichment: 23.64) as the most significantly associated with *MUC2* upregulation (Supplementary Figure S3). The cholinergic receptor nicotinic alpha 7 subunit (*CHRNA7*) gene (Spearman’s correlation: 0.456) (Supplementary Figure S4), which is involved in the molecular function described above, has also been reported to contribute to colon cancer progression [29], reflecting the complex regulation of CRC.

As they are joined together by disulfide bonds, MUC2 proteins can be depolymerized by reducing agents. Recently, disulfide bond-disrupting agents (DDAs), including RBF3 [30] and tcyDTDO [31], have been shown to kill breast tumors with acquired resistance to epidermal growth factor receptor (EGFR)/human epidermal growth factor receptor 2 (HER2) tyrosine kinase inhibitors. The resistance of these agents may in part be derived from the functional redundancy of EGFR family members, and DDAs can overcome such resistance by breaking down disulfide bonds that are required for the extracellular structural stability of those tyrosine kinases. However, whether DDAs can disrupt MUC2 to improve CCRT resistance needs further analysis. Using the Drug Repurposing Hub database (https://clue.io/repurposing) (accessed on 5 July 2021), we identified pranlukast as a clinically used drug to target MUC2. Pranlukast, a competitive antagonist of leukotriene C4 (LTC4), LTD4, and LTE4, is utilized in the management of asthma. It has been reported that LTD4 secreted from tumor-associated macrophages (M2-like) can promote colon cancer progression [32]. Intriguingly, LTD4 can also activate MUC2 via transcriptional regulation in colon adenocarcinoma [33]; nevertheless, whether pranlukast overcomes CCRT resistance through MUC2 downregulation in rectal cancer deserves further investigation.

## 5. Conclusions

In response to microenvironmental perturbations, the molecular characterization of rectal cancer in disease initiation, progression, metastasis, and treatment resistance may be divergent. In this study, we illustrated that upregulated MUC2 expression is connected with aggressive clinicopathological features, and MUC2 can independently act as an unfavorable prognostic indicator and a druggable target for rectal cancer patients receiving CCRT.

## Figures and Tables

**Table 1:** Summary of differentially expressed genes connected with maintenance of gastrointestinal epithelium (GO: 0030277) in relation to the response to CCRT in rectal carcinoma.

Probe	Comparison Log Ratio	Comparison *p*-Value	Gene Symbol	Gene Name	Biological Process	Molecular Function
204673_at	1.6574	0.0002	*MUC2*	mucin 2; oligomeric mucus/gel-forming	maintenance of gastrointestinal epithelium	extracellular matrix constituent; lubricant activity, extracellular matrix structural constituent
1565666_s_at	−0.0756	0.1125	*MUC6*	mucin 6; oligomeric mucus/gel-forming	maintenance of gastrointestinal epithelium	extracellular matrix structural constituent
214133_at	0.0009	0.9941	*MUC6*	mucin 6; oligomeric mucus/gel-forming	maintenance of gastrointestinal epithelium	extracellular matrix structural constituent
214676_x_at	0.1759	0.3632	*MUC3B*	mucin 3B; cell surface associated	maintenance of gastrointestinal epithelium	extracellular matrix constituent; lubricant activity
214898_x_at	0.2533	0.0837	*MUC3B*	mucin 3B; cell surface associated	maintenance of gastrointestinal epithelium	extracellular matrix constituent; lubricant activity
217117_x_at	0.0837	0.6297	*MUC3A*	mucin 3A; cell surface associated	maintenance of gastrointestinal epithelium	extracellular matrix constituent; lubricant activity

**Table 2:** Associations of MUC2 expression with clinicopathological variables in 172 rectal cancer patients undergoing neoadjuvant CCRT.

Parameter		No.	MUC2 Expression		*p*-Value
			Low Exp	High Exp.	
Gender	Male	108	52	56	0.528
	Female	64	34	30	
Age	<70	106	51	55	0.531
	≧70	66	35	31	
Pre-Tx tumor status (Pre-T)	T1-T2	81	48	33	0.022 *
	T3-T4	91	38	53	
Pre-Tx nodal status (Pre-N)	N0	125	72	53	0.001 *
	N1-N2	47	14	33	
Post-Tx tumor status (Post-T)	T1-T2	86	55	31	<0.001 *
	T3-T4	86	31	55	
Post-Tx nodal status (Post-N)	N0	123	73	50	<0.001 *
	N1-N2	49	13	36	
Vascular invasion	Absent	157	83	74	0.015 *
	Present	15	3	12	
Perineurial invasion	Absent	167	85	82	0.173
	Present	5	1	4	
Tumor regression grade	Grade 0–1	37	13	24	0.006 *
	Grade 2–3	118	59	59	
	Grade 4	17	14	3	

Tx, treatment; * statistically significant.

**Table 3:** Univariate log-rank analysis for important clinicopathological factors and MUC2 expression.

Parameter		No. of Case	DSS		LRFS		MeFS	
			No. of Event	*p*-Value	No. of Event	*p*-Value	No. of Event	*p*-Value
Gender	Male	108	20	0.9026	7	0.2250	17	0.3520
	Female	64	11		20		14	
Age	<70	106	19	0.8540	18	0.6615	20	0.7427
	≧70	66	12		9		11	
Pre-Tx tumor status (Pre-T)	T1–T2	81	10	0.0776	10	0.2261	11	0.1745
	T3–T4	91	21		17		20	
Pre-Tx nodal status (Pre-N)	N0	125	19	0.0711	15	0.0070 *	19	0.0973
	N1–N2	47	21		12		12	
Post-Tx tumor status (Post-T)	T1–T2	86	7	0.0006 *	7	0.0040 *	8	0.0033 *
	T3–T4	86	24		20		23	
Post-Tx nodal status (Post-N)	N0	123	21	0.5998	16	0.1320	20	0.4634
	N1–N2	49	10		11		11	
Vascular invasion	Absent	157	25	0.0184 *	21	0.0028 *	27	0.4470
	Present	15	6		6		4	
Perineurial invasion	Absent	167	29	0.2559	25	0.0940	30	0.9083
	Present	5	2		2		1	
Tumor regression grade	Grade 0–1	37	13	0.0038 *	10	0.0090 *	14	0.0006 *
	Grade 2–3	118	17		17		16	
	Grade 4	17	1		0		1	
Down stage after CCRT	Non-Sig.	150	29	0.1651	24	0.5961	30	0.0853
	Sig. (>=2)	22	2		3		1	
MUC2 expression	Low Exp.	86	3	<0.0001 *	5	<0.0001 *	6	<0.0001 *
	High Exp.	86	28		22		25	

DSS, disease-specific survival; LRFS, local recurrence-free survival; MeFS, metastasis-free survival; * statistically significant.

**Table 4:** Multivariate analysis.

Parameter	DSS			LRFS			MeFS		
	H.R	95% CI	*p*-Value	H.R	95% CI	*p*-Value	H.R	95% CI	*p*-Value
Tumor regression grade	1.869	0.932–3.759	0.078	2.247	1.049–4.807	0.037 *	2.326	1.156–4.673	0.018 *
MUC2 expression	9.507	2.794–32.353	<0.001 *	4.109	1.450–11.644	0.008*	4.023	1.616–10.013	0.003 *
Vascular invasion	1.621	0.645–4.073	0.304	2.094	0.757–5.796	0.155	-	-	-
Post-Tx tumor status (Post-T)	1.770	0.736–4.255	0.202	1.591	0.644–3.929	0.314	1.796	0.732–3.975	0.216
Pre-Tx nodal status (Pre-N)	-	-	-	1.479	0.617–3.545	0.381	-	-	-

DSS, disease-specific survival; LRFS, local recurrence-free survival; MeFS, metastasis-free survival; * statistically significant.

## Data Availability

The dataset analyzed in the current study (GSE35452) is available in the public transcriptome dataset from the Gene Expression Omnibus (GEO) database (National Center for Biotechnology Information, Bethesda, MD, USA).

## Supplementary Materials

The following are available online at https://www.mdpi.com/article/10.3390/jcm10143030/s1, Table S1. The top 200 genes positively correlated with MUC2; Table S2. The top 200 genes negatively correlated with MUC2; Figure S1. Correlations among *MUC2*, *REG4*, and *EREG* gene expression; Figure S2. The biological processes enriched in MUC2 upregulation and downregulation; Figure S3. The molecular functions enriched in *MUC2* upregulation and downregulation; Figure S4. Correlations between *MUC2* and *CHRNA7* gene expression.
