# Supplementary material for: Upregulated MUC2 Is an Unfavorable Prognostic Indicator for Rectal Cancer Patients Undergoing Preoperative CCRT
Source: J Clin Med. 2021 Jul 7;10(14):3030. doi: 10.3390/jcm10143030 (PMC8304358; doi:10.3390/jcm10143030)
Supplement: Supplementary file 1 [file jcm-10-03030-s001.zip › jcm-1212833_suppl v3.pptx]

## Slide 1
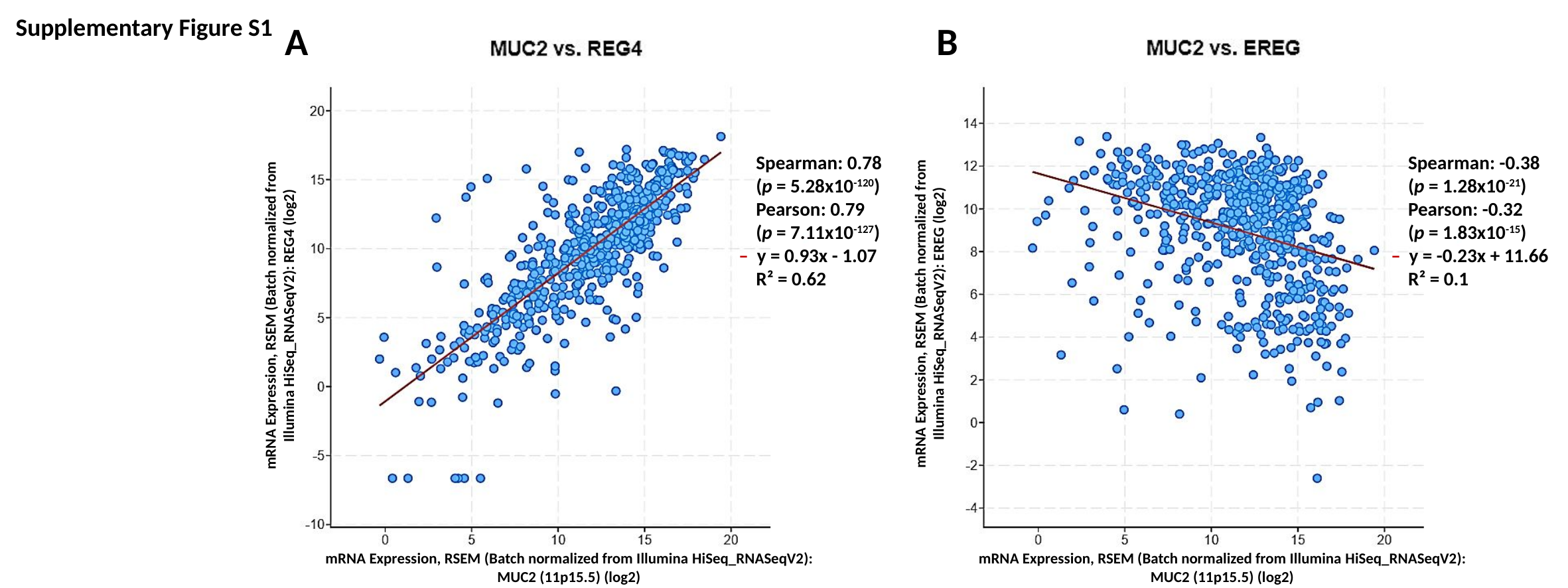

Supplementary Figure S1
A
B
mRNA Expression, RSEM (Batch normalized from Illumina HiSeq_RNASeqV2): EREG (log2)
mRNA Expression, RSEM (Batch normalized from Illumina HiSeq_RNASeqV2): REG4 (log2)
 Spearman: 0.78
 (p = 5.28x10-120)
 Pearson: 0.79
 (p = 7.11x10-127)
– y = 0.93x - 1.07
 R² = 0.62
 Spearman: -0.38
 (p = 1.28x10^-21^)
 Pearson: -0.32
 (p = 1.83x10^-15^)
– y = -0.23x + 11.66
 R² = 0.1
mRNA Expression, RSEM (Batch normalized from Illumina HiSeq_RNASeqV2): MUC2 (11p15.5) (log2)
mRNA Expression, RSEM (Batch normalized from Illumina HiSeq_RNASeqV2): MUC2 (11p15.5) (log2)
Supplementary Figure S1A–B. Correlations among MUC2, REG4, and EREG gene expression. The data were exported from TCGA database (n = 594) using the cBioPortal online platform.

## Slide 2
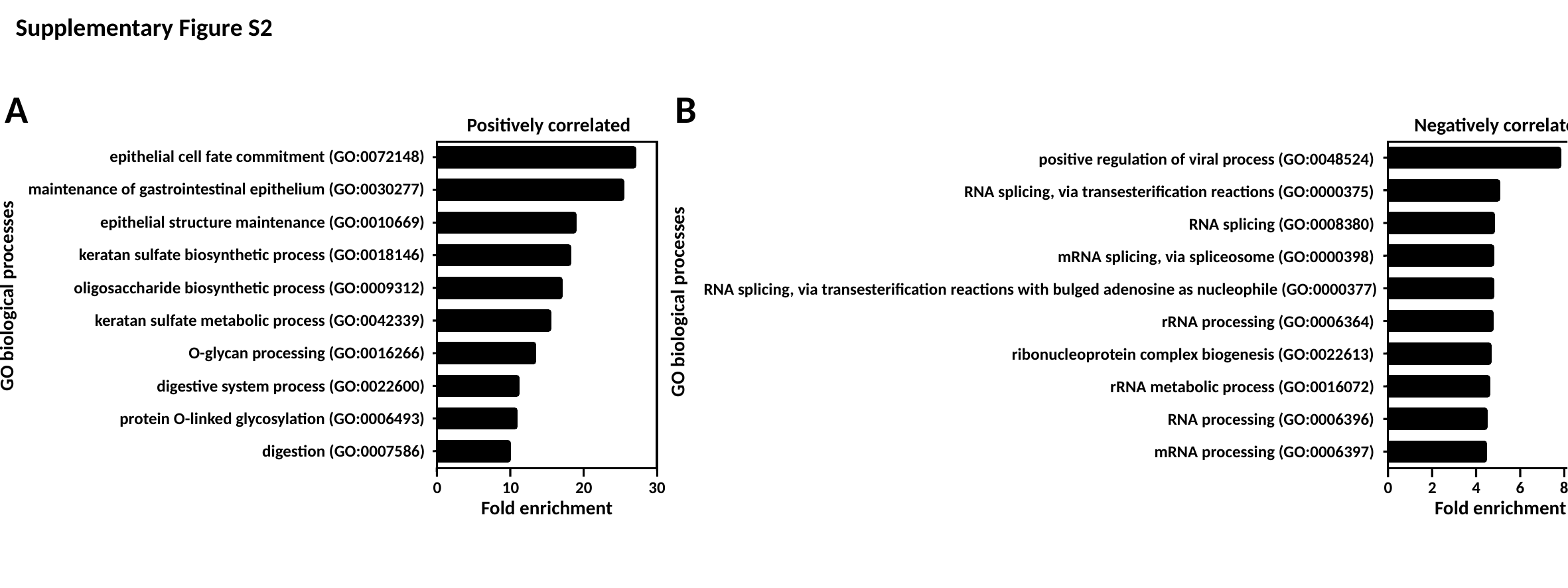

Supplementary Figure S2
A
B
Negatively correlated
positive regulation of viral process (GO:0048524)
GO biological processes
RNA splicing, via transesterification reactions (GO:0000375)
RNA splicing (GO:0008380)
mRNA splicing, via spliceosome (GO:0000398)
RNA splicing, via transesterification reactions with bulged adenosine as nucleophile (GO:0000377)
rRNA processing (GO:0006364)
ribonucleoprotein complex biogenesis (GO:0022613)
rRNA metabolic process (GO:0016072)
RNA processing (GO:0006396)
mRNA processing (GO:0006397)
0
2
4
6
8
10
Fold enrichment
Positively correlated
epithelial cell fate commitment (GO:0072148)
GO biological processes
maintenance of gastrointestinal epithelium (GO:0030277)
epithelial structure maintenance (GO:0010669)
keratan sulfate biosynthetic process (GO:0018146)
oligosaccharide biosynthetic process (GO:0009312)
keratan sulfate metabolic process (GO:0042339)
O-glycan processing (GO:0016266)
digestive system process (GO:0022600)
protein O-linked glycosylation (GO:0006493)
digestion (GO:0007586)
0
10
20
30
Fold enrichment
Supplementary Figure S2. The biological processes enriched in MUC2 upregulation and downregulation. The genes that were coexpressed with MUC2 in CRC from TCGA database (n = 594) were analyzed using the cBioPortal online platform (http://cbioportal.org) (accessed on 5 July 2021). The genes (top 200 transcripts) with either a (A) positive association or (B) negative association were further analyzed using PANTHER (http://pantherdb.org) (accessed on 5 July 2021) in accordance with biological processes and rated by fold enrichment for functional annotation.

## Slide 3
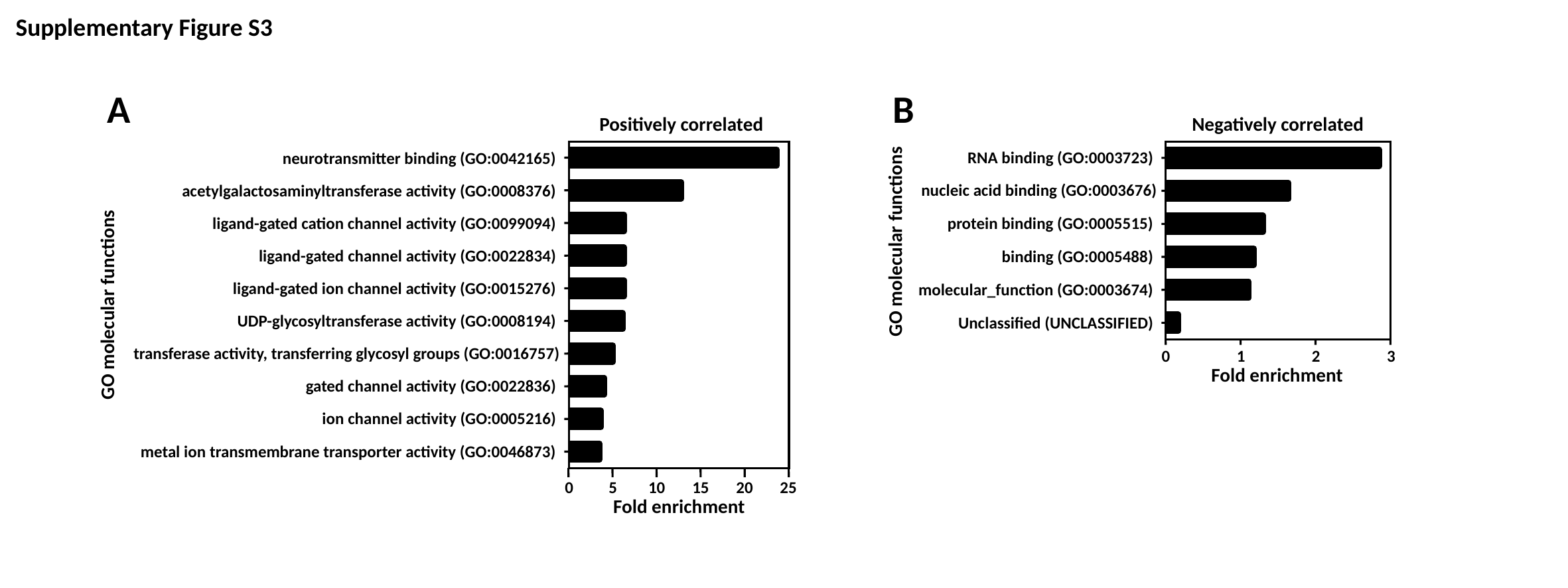

Supplementary Figure S3
A
B
Positively correlated
neurotransmitter binding (GO:0042165)
GO molecular functions
acetylgalactosaminyltransferase activity (GO:0008376)
ligand-gated cation channel activity (GO:0099094)
ligand-gated channel activity (GO:0022834)
ligand-gated ion channel activity (GO:0015276)
UDP-glycosyltransferase activity (GO:0008194)
transferase activity, transferring glycosyl groups (GO:0016757)
gated channel activity (GO:0022836)
ion channel activity (GO:0005216)
metal ion transmembrane transporter activity (GO:0046873)
0
5
10
15
20
25
Fold enrichment
GO molecular functions
Negatively correlated
RNA binding (GO:0003723)
nucleic acid binding (GO:0003676)
protein binding (GO:0005515)
binding (GO:0005488)
molecular_function (GO:0003674)
Unclassified (UNCLASSIFIED)
0
1
2
3
Fold enrichment
Supplementary Figure S3. The molecular functions enriched in MUC2 upregulation and downregulation. The genes (top 200 transcripts) with either a (A) positive association or (B) negative association were further analyzed using PANTHER (http://pantherdb.org) (accessed on 5 July 2021) according to molecular functions and rated by fold enrichment for functional annotation.

## Slide 4
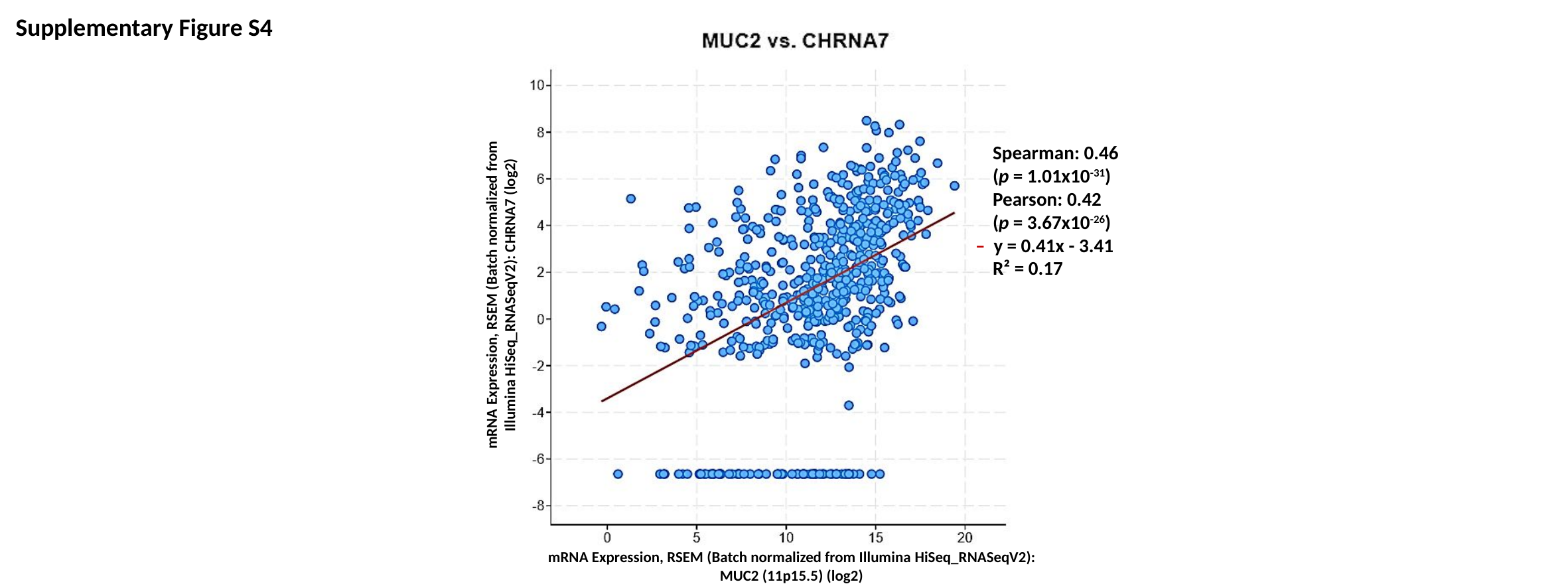

Supplementary Figure S4
mRNA Expression, RSEM (Batch normalized from Illumina HiSeq_RNASeqV2): CHRNA7 (log2)
 Spearman: 0.46
 (p = 1.01x10^-31^)
 Pearson: 0.42
 (p = 3.67x10^-26^)
– y = 0.41x - 3.41
 R² = 0.17
mRNA Expression, RSEM (Batch normalized from Illumina HiSeq_RNASeqV2): MUC2 (11p15.5) (log2)
Supplementary Figure S4. Correlations between MUC2 and CHRNA7 gene expression. The data were exported from TCGA database (n = 594) using the cBioPortal online platform.
