# Supplementary material for: Upregulated MUC2 Is an Unfavorable Prognostic Indicator for Rectal Cancer Patients Undergoing Preoperative CCRT
Source: J Clin Med. 2021 Jul 7;10(14):3030. doi: 10.3390/jcm10143030 (PMC8304358; doi:10.3390/jcm10143030)
Supplement: Supplementary file 1 [file jcm-10-03030-s001.zip › jcm-1212833_supplementary_tables.pdf]

Table S1. The top 200 genes positively correlated with MUC2.

Correlated Gene	Cytoband	Spearman's Correlation	*p*-Value	*q*-Value
FCGBP	19q13.2	0.835	7.52E-155	1.51E-150
B3GNT6	11q13.5	0.81	1.20E-138	1.20E-134
SPINK4	9p13.3	0.8	4.60E-133	3.07E-129
REG4	1p12	0.776	5.28E-120	2.64E-116
SPDEF	6p21.31	0.744	2.50E-105	1.00E-101
FER1L6	8q24.13	0.722	1.89E-96	6.32E-93
FAM177B	1q41	0.714	2.47E-93	7.06E-90
CHST5	16q23.1	0.695	1.83E-86	4.58E-83
ANO7	2q37.3	0.688	3.90E-84	8.67E-81
REP15	12p11.22	0.685	3.12E-83	6.24E-80
MUC4	3q29	0.681	9.10E-82	1.66E-78
ST6GALNAC1	17q25.1	0.674	1.43E-79	2.39E-76
HEPACAM2	7q21.2	0.673	3.19E-79	4.92E-76
FFAR4	10q23.33	0.671	1.53E-78	2.19E-75
ITLN1	1q23.3	0.669	6.59E-78	8.79E-75
ATOH1	4q22.2	0.667	1.63E-77	2.04E-74
ATP2A3	17p13.2	0.661	1.68E-75	1.98E-72
CREB3L1	11p11.2	0.657	1.90E-74	2.11E-71
TMEM61	1p32.3	0.656	3.01E-74	3.18E-71
CBFA2T3	16q24.3	0.646	3.16E-71	3.16E-68
LRRC26	9q34.3	0.645	5.40E-71	5.14E-68
NEURL1	10q24.33	0.645	7.34E-71	6.68E-68
KLF4	9q31.2	0.642	5.83E-70	5.07E-67
TPSG1	16p13.3	0.636	2.01E-68	1.68E-65
GALNT8	12p13.32	0.635	4.91E-68	3.93E-65
RAB26	16p13.3	0.619	7.10E-64	5.47E-61
ENTPD8	9q34.3	0.619	8.03E-64	5.95E-61
BEST2	19p13.13	0.618	1.37E-63	9.83E-61
GFI1	1p22.1	0.612	4.35E-62	3.01E-59
LINC00261	20p11.21	0.605	1.68E-60	1.12E-57
ZBTB7C	18q21.1	0.597	1.57E-58	1.02E-55
CLCA1	1p22.3	0.594	8.70E-58	5.44E-55
CTSE	1q32.1	0.591	5.71E-57	3.47E-54
ERN2	16p12.2	0.585	1.31E-55	7.71E-53
B3GALT5	21q22.2	0.581	9.45E-55	5.41E-52
CAPN9	1q42.2	0.581	1.15E-54	6.37E-52
MUC5B	11p15.5	0.576	1.54E-53	8.35E-51
MLPH	2q37.3	0.574	3.80E-53	2.00E-50
TOX	8q12.1	0.573	4.65E-53	2.39E-50
SLC4A4	4q13.3	0.572	8.12E-53	4.07E-50
RAP1GAP	1p36.12	0.571	2.04E-52	9.98E-50
KLK3	19q13.33	0.57	2.60E-52	1.24E-49
NBPF7	1p12	0.565	3.73E-51	1.74E-48
PTGER2	14q22.1	0.562	1.25E-50	5.70E-48
BCAS1	20q13.2	0.562	1.65E-50	7.34E-48
SGSM3	22q13.1	0.562	1.71E-50	7.45E-48
KLK1	19q13.33	0.56	3.58E-50	1.53E-47
ST6GALNAC6	9q34.11	0.558	1.07E-49	4.46E-47
ST3GAL4	11q24.2	0.556	1.98E-49	8.11E-47
IGFALS	16p13.3	0.556	2.20E-49	8.79E-47
TFF1	21q22.3	0.554	6.93E-49	2.72E-46
AQP3	9p13.3	0.553	1.02E-48	3.94E-46
VSIG2	11q24.2	0.55	3.53E-48	1.33E-45
SIDT1	3q13.2	0.548	9.04E-48	3.35E-45
SLC18A1	8p21.3	0.545	5.27E-47	1.92E-44
SLC22A23	6p25.2	0.544	5.71E-47	2.04E-44
KIF19	17q25.1	0.543	1.28E-46	4.50E-44
B4GALNT2	17q21.32	0.542	1.86E-46	6.42E-44
AGR2	7p21.1	0.542	2.03E-46	6.89E-44
KCNA6	12p13.32	0.533	1.12E-44	3.74E-42
NRAP	10q25.3	0.532	1.28E-44	4.19E-42
RASD1	17p11.2	0.532	1.43E-44	4.61E-42

B3GNT7	2q37.1|2q37.1	0.526	1.75E-43	5.57E-41
TSPAN1	1p34.1	0.526	2.11E-43	6.61E-41
ALDH1L1	3q21.3	0.525	3.73E-43	1.15E-40
SERPINA1	14q32.13	0.522	1.00E-42	3.04E-40
MMP28	17q12	0.519	3.58E-42	1.07E-39
ZG16	16p11.2	0.518	6.50E-42	1.91E-39
SCGB2A1	11q12.3	0.515	2.31E-41	6.71E-39
RHBDL3	17q11.2	0.511	9.24E-41	2.64E-38
GALNTL6	4q34.1	0.511	1.23E-40	3.46E-38
CANT1	17q25.3	0.51	1.34E-40	3.72E-38
LINC01550	14q32.2	0.508	3.51E-40	9.63E-38
ASRGL1	11q12.3	0.505	1.17E-39	3.17E-37
TRIM40	6p22.1	0.505	1.20E-39	3.22E-37
SLITRK6	13q31.1	0.505	1.44E-39	3.79E-37
FABP2	4q26	0.502	3.74E-39	9.72E-37
HYAL1	3p21.31	0.501	5.20E-39	1.32E-36
FAM83E	19q13.33	0.501	5.22E-39	1.32E-36
ITM2C	2q37.1	0.5	9.59E-39	2.40E-36
FAM189A2	9q21.12	0.499	1.61E-38	3.98E-36
NR3C2	4q31.23	0.498	1.98E-38	4.79E-36
IL1R2	2q11.2	0.496	4.24E-38	1.01E-35
CA2	8q21.2	0.496	4.39E-38	1.03E-35
CHST6	16q23.1	0.496	4.44E-38	1.03E-35
MB	22q12.3	0.494	1.09E-37	2.50E-35
GRIN1	9q34.3	0.492	2.41E-37	5.42E-35
LRP10	14q11.2	0.491	2.52E-37	5.60E-35
SLC35C1	11p11.2	0.488	9.50E-37	2.07E-34
CAPN5	11q13.5	0.487	1.55E-36	3.34E-34
TENT5A	6q14.1	0.486	2.40E-36	5.11E-34
TRIM7	5q35.3	0.482	9.07E-36	1.91E-33
NXPE2	11q23.2-q23.3	0.482	1.01E-35	2.11E-33
ANG	14q11.2	0.482	1.09E-35	2.25E-33
PLA2G2A	1p36.13	0.481	1.25E-35	2.55E-33
C2ORF88	2q32.2	0.48	2.26E-35	4.57E-33
RASD2	22q12.3	0.477	6.76E-35	1.35E-32
DLL1	6q27	0.474	1.48E-34	2.92E-32
SLC28A2	15q21.1	0.473	2.56E-34	5.02E-32
PAPSS2	10q23.2-q23.31	0.467	1.89E-33	3.67E-31
TNFRSF11A	18q21.33	0.467	2.09E-33	4.03E-31
AGR3	7p21.1	0.466	3.39E-33	6.41E-31
QSOX1	1q25.2	0.466	3.39E-33	6.41E-31
NKX2-2	20p11.22	0.465	4.56E-33	8.54E-31
STIM2	4p15.2	0.463	7.35E-33	1.36E-30
C4BPB	1q32.1	0.463	8.46E-33	1.55E-30
PTGDR2	11q12.2	0.463	1.01E-32	1.85E-30
DHRS9	2q31.1	0.462	1.43E-32	2.57E-30
CAMTA2	17p13.2	0.461	1.51E-32	2.69E-30
LPCAT4	15q14	0.461	1.57E-32	2.77E-30
SDR16C5	8q12.1	0.457	6.19E-32	1.09E-29
PLAC8	4q21.22	0.457	6.76E-32	1.18E-29
PTGER4	5p13.1	0.457	7.09E-32	1.22E-29
STYK1	12p13.2	0.457	7.24E-32	1.24E-29
CHRNA7	15q13.3	0.456	1.01E-31	1.72E-29
GPT	8q24.3	0.455	1.31E-31	2.21E-29
VWA3B	2q11.2	0.453	2.78E-31	4.63E-29
IL17REL	22q13.33	0.453	2.95E-31	4.89E-29
FFAR2	19q13.12	0.452	4.00E-31	6.51E-29
MUC1	1q22	0.451	5.12E-31	8.26E-29
TFF3	21q22.3	0.449	8.82E-31	1.41E-28
SI	3q26.1	0.553	1.17E-30	1.85E-28
GMPPB	3p21.31	0.446	3.04E-30	4.80E-28
KCNK6	19q13.2	0.445	3.47E-30	5.42E-28
GNE	9p13.3	0.444	6.16E-30	9.41E-28
CLCA4	1p22.3	0.443	6.89E-30	1.05E-27
RAB3B	1p32.3	0.442	9.23E-30	1.39E-27
DUSP4	8p12	0.441	1.33E-29	1.98E-27

CHRFAM7A	15q13.2	0.441	1.35E-29	1.98E-27
FOXA3	19q13.32	0.441	1.41E-29	2.07E-27
SCNN1A	12p13.31	0.441	1.42E-29	2.07E-27
SIDT2	11q23.3	0.441	1.65E-29	2.38E-27
PIGR	1q32.1	0.44	1.79E-29	2.56E-27
HID1	17q25.1	0.439	2.63E-29	3.74E-27
ARX	Xp21.3	0.439	2.92E-29	4.12E-27
TBC1D2	9q22.33	0.439	3.16E-29	4.43E-27
CATSPERB	14q32.12	0.438	3.71E-29	5.16E-27
BARX2	11q24.3	0.437	4.58E-29	6.31E-27
NPDC1	9q34.3	0.437	4.60E-29	6.31E-27
MADCAM1	19p13.3	0.437	5.76E-29	7.85E-27
ETHE1	19q13.31	0.435	1.05E-28	1.39E-26
NXPE1	11q23.2	0.434	1.32E-28	1.74E-26
VSTM5	11q21	0.434	1.54E-28	2.02E-26
RIMS3	1p34.2	0.433	1.97E-28	2.56E-26
PLA2G10	16p13.12	0.432	2.37E-28	3.06E-26
F3	1p21.3	0.432	2.54E-28	3.25E-26
SH3BGRL3	1p36.11	0.43	4.24E-28	5.33E-26
KIAA1324	1p13.3	0.43	4.72E-28	5.91E-26
RAB27B	18q21.2	0.43	5.23E-28	6.50E-26
TMEM246	9q31.1	0.429	6.23E-28	7.70E-26
GNA14	9q21.2	0.429	6.39E-28	7.85E-26
CA8	8q12.1	0.429	7.73E-28	9.44E-26
GCNT3	15q22.2	0.428	8.60E-28	1.04E-25
SLC44A4	6p21.33	0.428	9.43E-28	1.12E-25
ERGIC1	5q35.1	0.427	1.07E-27	1.26E-25
L1TD1	1p31.3	0.427	1.14E-27	1.33E-25
RETNLB	3q13.13	0.427	1.40E-27	1.63E-25
SLC9A1	1p36.11	0.427	1.43E-27	1.65E-25
PLCL2	3p24.3	0.426	1.87E-27	2.15E-25
SEC16A	9q34.3	0.425	2.28E-27	2.61E-25
LIMA1	12q13.12	0.425	2.35E-27	2.67E-25
GPAT3	4q21.23	0.425	2.44E-27	2.76E-25
TRPA1	8q21.11	0.424	2.77E-27	3.12E-25
EPHA4	2q36.1	0.424	3.04E-27	3.40E-25
BTNL8	5q35.3	0.423	4.42E-27	4.92E-25
SCIN	7p21.3	0.423	4.67E-27	5.15E-25
VSIG1	Xq22.3	0.422	6.39E-27	6.96E-25
RNASE4	14q11.2	0.421	8.67E-27	9.33E-25
IMPA2	18p11.21	0.42	1.02E-26	1.10E-24
TENT5C	1p12	0.42	1.04E-26	1.10E-24
VILL	3p22.2	0.42	1.20E-26	1.27E-24
SERINC4	15q15.3	0.419	1.29E-26	1.36E-24
TNFSF13	17p13.1	0.419	1.32E-26	1.39E-24
FAM114A1	4p14	0.419	1.42E-26	1.48E-24
ASPHD2	22q12.1	0.419	1.50E-26	1.56E-24
C2ORF72	2q37.1	0.419	1.57E-26	1.61E-24
LGALS4	19q13.2	0.418	1.78E-26	1.82E-24
MFSD2A	1p34.2	0.418	1.92E-26	1.95E-24
CKAP4	12q23.3	0.418	2.14E-26	2.16E-24
VPS53	17p13.3	0.417	2.36E-26	2.36E-24
ABHD3	18q11.2	0.416	3.19E-26	3.17E-24
REG1A	2p12	0.416	3.50E-26	3.47E-24
MRAP2	6q14.2	0.416	3.54E-26	3.49E-24
DMBT1	10q26.13	0.416	3.80E-26	3.73E-24
SHROOM3	4q21.1	0.416	3.84E-26	3.75E-24
MINK1	17p13.2	0.415	4.23E-26	4.11E-24
SLC37A1	21q22.3	0.415	4.60E-26	4.45E-24
KCNK1	1q42.2	0.414	6.13E-26	5.87E-24
LAMA1	18p11.31	0.414	6.61E-26	6.27E-24
ADGRF1	6p12.3|6	0.413	8.01E-26	7.56E-24
HSD17B2	16q23.3	0.413	8.94E-26	8.41E-24
PDE4D	5q11.2-q12.1	0.412	1.21E-25	1.13E-23
KLK12	19q13.41	0.411	1.40E-25	1.30E-23
CD55	1q32.2	0.411	1.47E-25	1.36E-23

VNN1	6q23.2	0.411	1.58E-25	1.45E-23
KLK11	19q13.41	0.411	1.68E-25	1.53E-23
TFF2	21q22.3	0.41	1.93E-25	1.75E-23
FAM174B	15q26.1	0.41	2.04E-25	1.84E-23
CD177	19q13.31	0.409	2.82E-25	2.52E-23
TOM1L2	17p11.2	0.409	3.14E-25	2.80E-23

Table S2. The top 200 genes negatively correlated with MUC2.

Correlated Gene	Cytoband	Spearman's Correlation	*p*-Value	*q*-Value
DPM1	20q13.13	−0.498	1.74E-38	**4.24E-36**
PFDN4	20q13.2	−0.493	1.66E-37	**3.77E-35**
RAE1	20q13.31	−0.489	7.23E-37	**1.59E-34**
MAPRE1	20q11.21	−0.452	4.00E-31	6.51E-29
METTL5	2q31.1	−0.444	5.02E-30	7.79E-28
EIF2S2	20q11.22	−0.444	5.68E-30	8.75E-28
RTF2	20q13.31	−0.442	1.12E-29	1.67E-27
TOMM34	20q13.12	−0.436	7.15E-29	9.67E-27
CSE1L	20q13.13	−0.436	8.05E-29	1.08E-26
CTNNBL1	20q11.23	−0.436	8.12E-29	1.08E-26
CCT6A	7p11.2	−0.432	2.56E-28	3.27E-26
ATP5F1E	20q13.32	−0.432	3.02E-28	3.83E-26
DCAF13	8q22.3	−0.428	7.95E-28	9.65E-26
TP53RK	20q13.12	−0.428	8.66E-28	1.04E-25
FAM217B	20q13.33	−0.428	1.03E-27	1.22E-25
OSER1	20q13.12	−0.423	4.68E-27	5.15E-25
HSPH1	13q12.3	−0.423	4.92E-27	5.38E-25
PDRG1	20q11.21	−0.421	7.53E-27	8.15E-25
POFUT1	20q11.21	−0.419	1.51E-26	1.56E-24
IFT52	20q13.12	−0.418	1.93E-26	1.96E-24
COMMD7	20q11.21	−0.414	6.05E-26	5.83E-24
RBIS	8q21.2	−0.414	6.44E-26	6.14E-24
DDX27	20q13.13	−0.412	1.34E-25	1.25E-23
DARS	2q21.3	−0.41	1.87E-25	1.70E-23
TATDN1	8q24.13	−0.409	2.70E-25	2.42E-23
TTPAL	20q13.12	−0.408	3.61E-25	3.16E-23
CIAO1	2q11.2	−0.408	3.66E-25	3.18E-23
SSB	2q31.1	−0.407	4.86E-25	4.16E-23
PPP1R3D	20q13.33	−0.407	5.71E-25	4.83E-23
DKC1	Xq28	−0.406	5.97E-25	5.00E-23
PCMTD2	20q13.33	−0.406	6.58E-25	5.44E-23
CWC15	11q21	−0.405	8.22E-25	6.66E-23
HSPE1	2q33.1	−0.405	8.99E-25	7.26E-23
EPDR1	7p14.1	−0.405	1.03E-24	8.23E-23
PDCD5	19q13.11	−0.403	1.60E-24	1.26E-22
NDRG3	20q11.23	−0.402	1.93E-24	1.50E-22
AAR2	20q11.23	−0.401	2.68E-24	2.05E-22
STK4	20q13.12	−0.4	3.54E-24	2.69E-22
NELFCD	20q13.32	−0.4	4.30E-24	3.23E-22
E2F6	2p25.1	−0.398	5.93E-24	4.43E-22
MOSPD1	Xq26.3	−0.398	6.79E-24	5.02E-22
PIPOX	17q11.2	−0.398	7.03E-24	5.15E-22
GID8	20q13.33	−0.397	7.64E-24	5.58E-22
PIGU	20q11.22	−0.397	9.02E-24	6.54E-22
NUFIP1	13q14.12	−0.395	1.67E-23	1.18E-21
CBX3	7p15.2	−0.395	1.73E-23	1.22E-21
JADE3	Xp11.3	−0.394	1.96E-23	1.38E-21
KRBOX4	Xp11.3	−0.394	2.00E-23	1.40E-21
SLC5A6	2p23.3	−0.394	2.09E-23	1.46E-21
REEP1	2p11.2	−0.393	2.36E-23	1.62E-21
FMR1	Xq27.3	−0.393	2.39E-23	1.64E-21
UTP6	17q11.2	−0.393	2.60E-23	1.78E-21
UBE2V1	20q13.13	−0.393	2.97E-23	2.02E-21
RBM39	20q11.22	−0.392	3.17E-23	2.14E-21
KIN	10p14	−0.391	4.97E-23	3.35E-21
ZC3H15	2q32.1	−0.391	5.17E-23	3.48E-21

WDR53	3q29	−0.39	5.64E-23	3.78E-21
ZNF251	8q24.3	−0.389	8.70E-23	5.77E-21
YTHDF1	20q13.33	−0.388	1.01E-22	6.63E-21
RHEB	7q36.1	−0.388	1.04E-22	6.77E-21
LY6G6D	6p21.33	−0.388	1.07E-22	6.98E-21
POLR2K	8q22.2	−0.388	1.16E-22	7.53E-21
NFS1	20q11.22	−0.387	1.30E-22	8.25E-21
STAU1	20q13.13	−0.387	1.54E-22	9.64E-21
AMACR	5p13.2	−0.386	1.78E-22	1.10E-20
TCFL5	20q13.33	−0.386	1.94E-22	1.19E-20
GNPDA1	5q31.3	−0.385	2.07E-22	1.26E-20
PRELID3B	20q13.32	−0.385	2.35E-22	1.41E-20
WDSUB1	2q24.2	−0.385	2.61E-22	1.56E-20
ZPR1	11q23.3	−0.384	2.85E-22	1.69E-20
PNO1	2p14	−0.384	2.89E-22	1.71E-20
CTSV	9q22.33	−0.384	3.10E-22	1.82E-20
NOL10	2p25.1	−0.384	3.21E-22	1.88E-20
GTF2F2	13q14.12-q14.13	−0.384	3.25E-22	1.90E-20
RNF219	13q31.1	−0.384	3.25E-22	1.90E-20
PHF20	20q11.22-q11.23	−0.384	3.29E-22	1.91E-20
UTP14A	Xq26.1	−0.383	3.55E-22	2.05E-20
WBP4	13q14.11	−0.383	3.77E-22	2.17E-20
LAMP2	Xq24	−0.383	4.12E-22	2.36E-20
GLS	2q32.2	−0.383	4.34E-22	2.48E-20
ACTR5	20q11.23	−0.383	4.47E-22	2.53E-20
YWHAB	20q13.12	−0.383	4.47E-22	2.53E-20
METTL6	3p25.1	−0.382	5.14E-22	2.90E-20
ZSWIM3	20q13.12	−0.381	6.26E-22	3.50E-20
RAB22A	20q13.32	−0.381	7.02E-22	3.92E-20
UQCC1	20q11.22	−0.381	7.06E-22	3.92E-20
SLC2A12	6q23.2	−0.381	7.21E-22	4.00E-20
SF3B6	2p23.3	−0.38	7.90E-22	4.37E-20
OSGIN2	8q21.3	−0.38	8.04E-22	4.44E-20
ADNP	20q13.13	−0.38	8.37E-22	4.61E-20
YAP1	11q22.1	−0.38	9.07E-22	4.96E-20
PPP1R2	3q29	−0.379	1.06E-21	5.76E-20
RPIA	2p11.2	−0.379	1.15E-21	6.26E-20
EREG	4q13.3	−0.379	1.28E-21	6.91E-20
TIMM8A	Xq22.1	−0.378	1.50E-21	8.00E-20
CSNK2A2	16q21	−0.377	1.76E-21	9.32E-20
MRPS31	13q14.11	−0.377	1.79E-21	9.46E-20
PHACTR3	20q13.32-q13.33	−0.377	1.80E-21	9.47E-20
DYNLRB1	20q11.22	−0.377	2.09E-21	1.09E-19
MITD1	2q11.2	−0.376	2.37E-21	1.23E-19
GPN1	2p23.3	−0.376	2.80E-21	1.45E-19
ANXA9	1q21.3	−0.376	2.84E-21	1.47E-19
FUNDC1	Xp11.3	−0.375	3.15E-21	1.61E-19
SLC4A1AP	2p23.3	−0.375	3.39E-21	1.72E-19
ETNK2	1q32.1	−0.375	3.67E-21	1.86E-19
COPS8	2q37.3	−0.373	5.18E-21	2.60E-19
NORAD	20q11.23	−0.373	5.46E-21	2.74E-19
ORC3	6q15	−0.373	5.54E-21	2.77E-19
TTI1	20q11.23	−0.373	5.81E-21	2.90E-19
DPY30	2p22.3	−0.372	6.42E-21	3.18E-19
HIKESHI	11q14.2	−0.372	6.71E-21	3.31E-19
TSPAN6	Xq22.1	−0.372	7.37E-21	3.61E-19
TPD52L2	20q13.33	−0.372	8.22E-21	4.02E-19
CLNS1A	11q14.1	−0.371	1.03E-20	4.96E-19
GGH	8q12.3	−0.369	1.55E-20	7.38E-19
ELOC	8q21.11	−0.369	1.61E-20	7.67E-19
DDX10	11q22.3	−0.367	2.29E-20	1.08E-18
BCL11A	2p16.1	−0.367	2.87E-20	1.35E-18
DSN1	20q11.23	−0.366	3.12E-20	1.46E-18
SMYD5	2p13.2	−0.366	3.34E-20	1.56E-18
NODAL	10q22.1	−0.366	3.50E-20	1.62E-18
CDK5RAP1	20q11.21	−0.365	3.80E-20	1.75E-18

MPHOSPH10	2p13.3	−0.365	4.09E-20	1.88E-18
WNT11	11q13.5	−0.365	4.62E-20	2.11E-18
SCML1	Xp22.13	−0.365	4.85E-20	2.19E-18
PSMA7	20q13.33	−0.364	5.25E-20	2.36E-18
KRT23	17q21.2	−0.364	5.32E-20	2.38E-18
ATIC	2q35	−0.364	5.84E-20	2.59E-18
COIL	17q22	−0.364	6.17E-20	2.73E-18
NCOA5	20q13.12	−0.363	6.52E-20	2.87E-18
COMMD6	13q22.2	−0.363	6.65E-20	2.93E-18
PIP4P2	8q21.3	−0.363	7.12E-20	3.13E-18
NEU1	6p21.33	−0.363	7.90E-20	3.44E-18
NDUFAF7	2p22.2	−0.362	8.07E-20	3.51E-18
VMA21	Xq28	−0.362	8.92E-20	3.87E-18
RAB5IF	20q11.23	−0.362	9.33E-20	4.03E-18
PSPH	7p11.2	−0.362	9.45E-20	4.08E-18
PDCL3	2q11.2	−0.362	9.55E-20	4.11E-18
RPRD1B	20q11.23	−0.362	1.00E-19	4.29E-18
CHMP4B	20q11.22	−0.361	1.03E-19	4.40E-18
PRMT3	11p15.1	−0.361	1.16E-19	4.90E-18
GEMIN6	2p22.1	−0.361	1.16E-19	4.92E-18
FOXQ1	6p25.3	−0.361	1.22E-19	5.14E-18
CAMKMT	2p21	−0.361	1.25E-19	5.26E-18
PUS10	2p16.1-p15	−0.361	1.31E-19	5.48E-18
WDR35	2p24.1	−0.36	1.33E-19	5.56E-18
BRD7	16q12.1	−0.36	1.47E-19	6.09E-18
CHCHD7	8q12.1	−0.36	1.58E-19	6.55E-18
STX16	20q13.32	−0.359	2.05E-19	8.45E-18
ENOX2	Xq26.1	−0.359	2.06E-19	8.47E-18
NCBP2	3q29	−0.358	2.40E-19	9.77E-18
NDUFAF6	8q22.1	−0.358	2.41E-19	9.77E-18
R3HDML	20q13.12	−0.358	2.72E-19	1.10E-17
DUS4L	7q22.3	−0.358	2.74E-19	1.10E-17
COPS5	8q13.1	−0.357	3.06E-19	1.23E-17
STAMBP	2p13.1	−0.357	3.41E-19	1.36E-17
PNPT1	2p16.1	−0.356	3.54E-19	1.41E-17
WDR12	2q33.2	−0.356	3.60E-19	1.43E-17
SLC19A3	2q36.3	−0.356	3.75E-19	1.48E-17
MRE11	11q21	−0.356	3.92E-19	1.54E-17
SMARCE1	17q21.2	−0.356	4.09E-19	1.60E-17
UTP23	8q24.11	−0.356	4.44E-19	1.73E-17
ANKRD46	8q22.3	−0.355	4.48E-19	1.74E-17
VAPB	20q13.32	−0.355	4.82E-19	1.87E-17
MRPL48	11q13.4	−0.355	5.27E-19	2.04E-17
HSF2	6q22.31	−0.354	5.83E-19	2.24E-17
USP39	2p11.2	−0.354	6.33E-19	2.43E-17
LTV1	6q24.2	−0.354	6.58E-19	2.52E-17
IMMP2L	7q31.1	−0.354	6.85E-19	2.62E-17
RPF2	6q21	−0.353	7.49E-19	2.83E-17
CYB5B	16q22.1	−0.353	7.58E-19	2.86E-17
CEL	9q34.13	−0.352	9.48E-19	3.56E-17
NUDCD1	8q23.1	−0.352	9.66E-19	3.62E-17
CAB39L	13q14.2	−0.352	1.13E-18	4.21E-17
HABP4	9q22.32	−0.352	1.16E-18	4.30E-17
C11ORF95	11q13.1	−0.352	1.17E-18	4.33E-17
VAV3	1p13.3	−0.351	1.29E-18	4.79E-17
HSPBAP1	3q21.1	−0.351	1.31E-18	4.85E-17
CSTF1	20q13.2-q13.31	−0.35	1.78E-18	6.49E-17
GRPR	Xp22.2	−0.349	1.90E-18	6.92E-17
RNF114	20q13.13	−0.349	1.98E-18	7.21E-17
GPSM2	1p13.3	−0.349	2.07E-18	7.49E-17
HSPD1	2q33.1	−0.349	2.16E-18	7.83E-17
LDLRAD3	11p13	−0.349	2.21E-18	7.97E-17
UXS1	2q12.2	−0.349	2.24E-18	8.07E-17
NKD1	16q12.1	−0.349	2.39E-18	8.58E-17
RALGAPB	20q11.23	−0.348	2.46E-18	8.80E-17
RBMX2	Xq26.1	−0.348	2.49E-18	8.91E-17

MSANTD4	11q22.3	−0.348	2.55E-18	9.11E-17
MED30	8q24.11	−0.348	2.61E-18	9.31E-17
CMSS1	3q12.1	−0.348	3.02E-18	1.07E-16
SUMO1P3	1q23.2	−0.347	3.16E-18	1.12E-16
NSMCE2	8q24.13	−0.347	3.33E-18	1.17E-16
ZC3H8	2q14.1	−0.347	3.39E-18	1.19E-16
FAM192A	16q13	−0.347	3.78E-18	1.33E-16
UCHL3	13q22.2	−0.346	4.38E-18	1.53E-16
SYS1	20q13.12	−0.346	4.46E-18	1.56E-16
C7ORF25	7p14.1	−0.346	4.75E-18	1.65E-16
APLF	2p13.3	−0.344	6.33E-18	2.17E-16
ATP6AP2	Xp11.4	−0.344	6.60E-18	2.26E-16
